# Dissecting recurrent waves of pertussis across the boroughs of London

**DOI:** 10.1371/journal.pcbi.1009898

**Published:** 2022-04-14

**Authors:** Arash Saeidpour, Shweta Bansal, Pejman Rohani

**Affiliations:** 1 Odum School of Ecology, University of Georgia, Athens, Georgia, United States of America; 2 Department of Biology, Georgetown University, Washington, D.C., United States of America; 3 Department of Infectious Diseases, College of Veterinary Medicine, University of Georgia, Athens, Georgia, United States of America; 4 Center for Influenza Disease & Emergence Research (CIDER), Athens, Georgia, United States of America; University of Notre Dame, UNITED STATES

## Abstract

Pertussis has resurfaced in the UK, with incidence levels not seen since the 1980s. While the fundamental causes of this resurgence remain the subject of much conjecture, the study of historical patterns of pathogen diffusion can be illuminating. Here, we examined time series of pertussis incidence in the boroughs of Greater London from 1982 to 2013 to document the spatial epidemiology of this bacterial infection and to identify the potential drivers of its percolation. The incidence of pertussis over this period is characterized by 3 distinct stages: a period exhibiting declining trends with 4-year inter-epidemic cycles from 1982 to 1994, followed by a deep trough until 2006 and the subsequent resurgence. We observed systematic temporal trends in the age distribution of cases and the fade-out profile of pertussis coincident with increasing national vaccine coverage from 1982 to 1990. To quantify the hierarchy of epidemic phases across the boroughs of London, we used the Hilbert transform. We report a consistent pattern of spatial organization from 1982 to the early 1990s, with some boroughs consistently leading epidemic waves and others routinely lagging. To determine the potential drivers of these geographic patterns, a comprehensive parallel database of borough-specific features was compiled, comprising of demographic, movement and socio-economic factors that were used in statistical analyses to predict epidemic phase relationships among boroughs. Specifically, we used a combination of a feed-forward neural network (FFNN), and SHapley Additive exPlanations (SHAP) values to quantify the contribution of each covariate to model predictions. Our analyses identified a number of predictors of a borough’s historical epidemic phase, specifically the age composition of households, the number of agricultural and skilled manual workers, latitude, the population of public transport commuters and high-occupancy households. Univariate regression analysis of the 2012 epidemic identified the ratio of cumulative unvaccinated children to the total population and population of Pakistan-born population to have moderate positive and negative association, respectively, with the timing of epidemic. In addition to providing a comprehensive overview of contemporary pertussis transmission in a large metropolitan population, this study has identified the characteristics that determine the spatial spread of this bacterium across the boroughs of London.

## Author summary

Recent years have witnessed a resurgence of pertussis, a vaccine preventable bacterial disease, in countries with high estimated immunization rates. Here, to understand the timing of pertussis epidemics in different populations, we employed signal processing techniques to analyze a rich dataset of weekly incidence across the boroughs of London from 1982 to 2013. We observed 4-year epidemic cycles with a distinctly consistent spatial organization across the boroughs from 1982 to 1990, with some boroughs typically ahead of the wave and others consistently lagging. We identified putative demographic and socio-economic mechanisms that determined this spatial organization of these outbreaks using an interpretable machine learning approach. The reemergence of pertussis from 2006 onward, on the contrary, did not exhibit regular epidemic waves, instead there was a transition from spatially organized waves in the 1980s to a largely unstructured mosaic in the resurgence era.

## Introduction

Understanding the spatial dynamics and diffusion of infectious diseases is increasingly recognized as an important component in shedding light on their underlying epidemiology and developing effective control policies [1–6]. This is true both for emerging infectious diseases whose spatial trajectory needs to be predicted [7–11] and those vaccine-preventable infectious diseases whose elimination is stymied by the stubborn mosaic of under-vaccination [12–16] or asynchronous metapopulation persistence [17, 18]. Key to this effort has been attempting to understand the drivers of the observed spatial ecology of these systems, including the impact of host movement [5, 11, 19, 20] and workflows [7], urban-rural gradients [1], regional variation in climate seasonality [4, 21–23] or population demography [3] and the impact of road networks [24]. These studies have identified insights that may, in principle, inform control policies including the implementation of spatially synchronised pulsed vaccination to increase the likelihood of elimination when epidemics are asynchronous [17, 25, 26], quantifying the impacts of border closures [9] and geographic barriers [27, 28] on transmission.

Here, we examined the spatial ecology of recurrent pertussis epidemics in the boroughs of London from 1982–2013. Pertussis is a bacterial pathogen once considered a disease of childhood [16, 29, 30] and whose recent resurgence in a number of countries with high estimated immunization coverage has generated a great deal of debate [31–37]. Prior studies of the spatial epidemiology of pertussis have demonstrated a range of distinct patterns [16]. Dynamics in the pre-vaccine era (prior to 1957) in the cities of England & Wales were shown to be characterized by spatial asynchrony, which gave way to largely synchronous 4-year epidemics in the early whole cell (wP) vaccination era [18]. Similarly, studying incidence reports from provinces of Thailand, Blackwood *et al*. [38] documented regular annual pertussis outbreaks that were highly in-phase with each other. Finally, a study of pertussis incidence in US states found evidence of traveling waves in the early years of wP vaccination, 1950s-1960s [5, 39]. These waves spread inwards from two distinct regional foci located at each coast, one in the Northeast and the other in the Northwest [5]. Comparable analyses of pertussis data for the US since its re-emergence over the past four decades [39, 40] did not identify any discernible spatial organization [5].

To dissect the spatial epidemiology of pertussis at a finer spatial scale than these previous analyses, we took advantage of a rich and well-documented disease incidence dataset collected by Public Health England (PHE) [41–44]. These weekly *Notifications of Infectious Diseases (NOIDs)* data from 1982 to 2013 confirmed the evolving nature of pertussis epidemiology. Over this period, there were three distinct stages in the incidence of pertussis in the boroughs of London: (i) a period of declining trends with four-year inter-epidemic cycles from 1982 to 1994, (ii) a period characterized by a deep trough in incidence from 1994 until 2006, and (iii) a subsequent reemergence of the disease leading to a large outbreak in 2012. In the first phase, increasing infant wP vaccination coverage was associated with a drop in incidence, an increase in the age of reported infected and a higher frequency of weeks with no pertussis notifications [45]. These data also clearly identified regular 4-year epidemics in London boroughs, with surprisingly consistent phase relationships among locations through time. In contrast, the reemergence of pertussis from 2006 onward did not exhibit periodic epidemic waves.

We sought to identify putative mechanisms that determined the spatial organization of these outbreaks using an interpretable machine learning approach. We integrated the NOIDs data with a comprehensive database of borough-specific demographic, movement and socio-economic factors to carry out statistical analyses of epidemic phase relationships among the boroughs.

## Materials and methods

### Incidence data

Weekly pertussis incidence for the 32 boroughs of Greater London were obtained from *Notifications of Infectious Diseases* (NOIDs), spanning 1982–2013. Note that prior to 1994, case confirmations were based solely on the isolation and culture of the causative bacterium, *Bordetella pertussis* [46]. Since then, an enhanced surveillance system with epidemiological follow up for laboratory confirmation of cases has been established, with confirmation of clinically suspected cases achieved by culture, use of PCR to detect bacterial DNA from nasopharyngeal samples or antibody detection performed on oral fluid or serum [41, 46].

### Geographic, demographic and socioeconomic variables

We compiled a comprehensive set of demographic and socio-economic factors from aggregated census data collected in 1981, 1991, 2001 and 2011 at the borough level (see Table 1). Since the census is carried out every 10 years, covariate values falling in-between census years were obtained by interpolation using B-splines, as shown in S1 Fig and mapped in S2–S5 Figs.

**Table 1:** Description of geographic and census variables used in this study.

Variable	Definition	Source
Number of households with no children	-	[58]
Number of households with children aged 0–4	-	[58]
Number of households with children aged 5–16	-	[58]
Born in Africa	Number of people born in Africa	[58]
Born in Caribbean	Number of people born in Caribbean	[58]
Born in India	Number of people born in India	[58]
Born in Pakistan	Number of people born in Pakistan	[58]
Pres. & Res. comm. estbls.	Number of people present and all usual residents in communal establishments	[58]
Households with >1.5 PPR	Number of households with >1.5 people per room	[58]
Not self contained houses	Number of not self-contained housesholds (self-containment means that all rooms, including the kitchen, bathroom and toilet are behind a door (but not necessarily a single door) only that household can use.)	[58]
SEG 1–4	Number of people in SEG*1 to 4 (Employers, Managers and Professional Workers)	[58]
SEG 8,9,12	Number of people in SEG*8,9 and 12 (Skilled Manual Workers)	[58]
SEG 7–10	Number of people in SEG*7 to 10 (Skilled Manual Workers)	[58]
SEG 11	Number of people in SEG*11 (Unskilled Manual Workers)	[58]
SEG 13–15	Number of people in SEG*13 to 15 (Farmers and Agricultural Workers)	[58]
SEG 16–17	Number of people in SEG*16 and 17 (Armed Forces and Not Stated)	[58]
Travel public	Number of people who use means of public transportation to travel to work (British Rails, other rail, bus)	[58]
Travel other modes	Number of people who use other means of transportation to travel to work (car, motorcycle, bicycle, other)	[58]
Inland Area (Hectares)	Borough’s inland area (excluding bodies of water)	[59]
Longitude	Longitude of borough’s geometric centroid	[60]
Latitude	Latitude of borough’s geometric centroid	[60]

* Socio-economic group, see [52].

#### Household age composition

Past work has indicated a shift in the age distribution of cases in England and Wales from 1982 to 1994 [45]. While children and infants accounted for the majority of cases in the early years of this era, there was higher incidence in adults and adolescents in later years [41]. In order to examine the potential contribution of different age groups to epidemic phase lags, we complied data on household age composition from the census data, which provide *Number of households with no children*, *Number of households with children aged 0–4* and *Number of households with children age 5–16* for each borough. We used these data as predictor features in our statistical models.

#### Movement and geographic features

It has been shown that correlation between disease prevalence in connected populations is dependent on the flow of individuals between them [7, 20, 47]. This prompted us to examine the potential relationship between public and private commuter travel patterns and epidemic phase lags. We have incorporated the sum of all public (British rail, other rail, bus) and private (car, motorcycle, bicycle) modes of travel to work (*Travel public* and *Travel private*, respectively). We were also motivated to study the potential impact of gravitational coupling, whereby the magnitude of epidemiological exchange between two locations is determined by their distance and respective population sizes. Previous studies have identified a role of gravity models in the epidemics of measles in England and Wales [1, 19], waves of influenza in the US [7], the spread of ebola in West Africa [9] and hierarchies of dengue haemorrhagic fever in Thailand [48]. Thus, to allow for this mechanism, the *Latitude* and *Longitude* of the geographical centroid of each borough and *Inland Area* (Hectares) were added as geographical factors.

#### Socio-economic features

We sought to examine whether socio-economic heterogeneity among boroughs can explain epidemic phase relationships. Thus, we included the Socio-Economic Group *(SEG)*, which is a classification based on the employment status and occupation of individuals. According to the UK census, the population of each borough is stratified into 6 aggregate *SEG* classes, as shown in Table 1. Additionally, the number of people present and all usual residents in communal establishments outside the medical and care sector (*Pres. & Res. comm. estbls*.) and the number of not self-contained houses (*Not self-contained houses*) were also considered as potential indicators of economic status. To investigate possible statistical association between the composition of population ethnicity and epidemic timing of outbreaks, the number of individuals by place of birth for four major immigrant populations (Africa, Caribbean, India and Pakistan) were considered.

#### Household characteristics

The role of household characteristics has been identified in spatial spread of influenza across Europe [49] and pertussis epidemics in England and Wales [50] and the Netherlands [51]. To evaluate the combined effects of household occupancy, the Number of households with > 1.5 people per room (*Households with > 1.5 PPR*) was included among predictor variables. As shown in S5 Fig, we identified pairwise linear associations between a number of the described variables (see [52] for more details about the census).

#### Vaccine coverage estimates

Intuitively, a potentially important factor in shaping pertussis epidemics, especially the timing of outbreaks, may be susceptible births [53–55], which has been previously shown to determine the seasonal outbreaks of rotavirus in the US [3]. We have, however, been unable to obtain borough-specific routine immunization coverage estimates spanning the 1982–1996 period. It is plausible that some of our socio-economic features serve as a proxy for vaccination coverage [56].

We obtained immunization rates from the former Health Protection Agency (HPA) starting in 1997–98, which was the earliest available data at the borough level [57]. We carried out statistical analyses to examine potential association between these vaccine coverage data and epidemic phase lags among boroughs.

### Epidemic time lag analysis

To analyze data from the first phase (1982–1994), weekly case count time series from each borough were first square rooted to stabilize the variance and then normalized to have zero mean. Normalized time series were then zero-padded to the next higher power of two to minimize edge effects [61]. We applied the continuous Morlet wavelet transform on the normalized time series to determine the range of dominant periods of the epidemic wave. We then filtered the time series with a band-pass filter corresponding to the dominant period and applied the Hilbert transform to filtered time series to extract the instantaneous phase angles. An important advantage of the Hilbert transform is that phase angles can be extracted for an arbitrary broad-band signal, where signal power is mainly distributed in a specified range of frequencies (see [62] for a comparison of different signal processing methods for quantification of phase synchrony). This contrasts with the continuous wavelet transform, which traditionally has been used for decomposition of ecological time series [1, 5]. We extracted the instantaneous phase angle of each borough at three time points (the outbreaks of 1982, 1986, 1990) and defined the phase lag of a given borough at each snapshot as the difference between the borough’s instantaneous phase angle and the mean phase angle of all boroughs at the corresponding instant (the pre-processing and transformation steps are illustrated in S6 Fig).

Wavelet analysis of resurgence incidence data (2006–2013) did not exhibit periodic epidemic waves across the boroughs. To examine the spatial ecology of the 2012 epidemic, therefore, we used Kernel density estimation (KDE) [63] to quantify the epidemic time lag as the mean of a Gaussian distribution.

### Fade-out frequency profile

We used a 4-year sliding window to calculate the average number of fade-outs per year. Three consecutive weeks with no reported cases was counted as one fade-out [64–66]. The total number of fade-outs in the counting window were then divided by four to obtain the average number of weekly fade-outs. The counting window is successively shifted 6-months ahead to count the number of fade-outs through time.

### Feature importance

To examine the potential statistical association between the epidemic phase lag of boroughs and the predictor variables, we employed different regression methods with varying levels of complexity. The marginal contribution of each variable to the model predictions is deemed as feature importance. The variables are standardized to have mean zero and unit variance. All the regression models are fitted to the standardized data.

We first performed univariate regression to investigate the individual importance of each variable. This was followed by ordinary least squares (OLS) multivariate linear regression analysis to examine the combined effects of variables. Multivariate linear analysis was repeated with L1 (lasso) and L2 (ridge) regularizations [67]. Finally, to capture any potential nonlinear effects, we used a feed-forward neural network model (FFNN). The model was implemented in the ‘Keras’ [68] Python package with a ‘TensorFlow’ [69] backend. We constrained our architecture search to a maximum of 3 hidden layers and up to 64 nodes per layer with *tanh* activation. Dropout regularization was used to prevent overfitting [70]. We used Adam optimizer [71] and limited the number of epochs to 2000.

For model evaluation and hyperparameter tuning, we randomly split the data into 64 training samples and 32 test samples. The regularization parameter for lasso and ridge regression analysis was optimized via 10-fold cross validation over the training samples. For the FFNN model hyperparameter optimization, we randomly selected 16 samples out of 64 training samples as the validation set. The model’s hyperparameters were tuned via a grid search over the validation set.

Upon training the FFNN model, we used the SHapley Additive exPlanations (SHAP) method [72, 73] to calculate the marginal contribution of each feature to the predicted value by the model. SHAP is a novel, model-agnostic method for measuring local interactions [74], rooted in Shapley values proposed in the theory of cooperative games [75]. Model-agnostic methods provide a *post hoc* interpretation for arbitrary machine learning models by treating them as black-boxes, where interpretation is obtained by fitting an explainable model to the predictions of the black-box model, or measuring the variation in the black-box model predictions with small perturbations in inputs or both [74]. These methods allow for local interpretation of a single input instance or a group of input data (See S1 Text for more information on SHAP).

## Results

### Epidemiological characteristics

**Fig 1:**
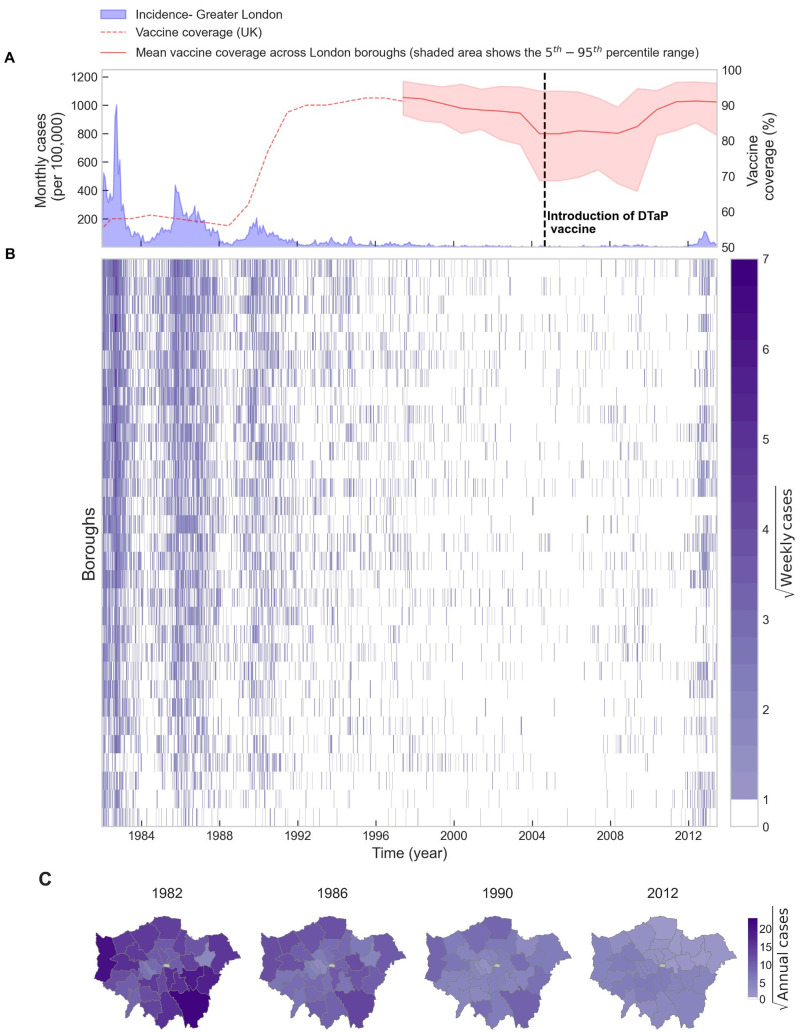
Evolution of pertussis incidence in Greater London. (A) Time series of monthly reported cases in Greater London and annual vaccine coverage rate in the UK (B) Square root of weekly cases of pertussis in London boroughs (C) Choropleth maps of square root of annual cases in the London boroughs at four time snapshots. Map base layers are obtained from London Datastore (https://data.london.gov.uk/dataset/statistical-gis-boundary-files-london) and are available from https://data.london.gov.uk/download/statistical-gis-boundary-files-london/9ba8c833-6370-4b11-abdc-314aa020d5e0/statistical-gis-boundaries-london.zip (The digital boundary file contains Office for National statistics data Crown copyright and database (2012) and contains Ordnance Survey data Crown copyright and database (2012)).

We illustrate pertussis incidence in Greater London during the study period in Fig 1A. Data from the first phase (1982–1994) are characterized by regular 4-year epidemics with a decreasing trend in the amplitude. Routine vaccination coverage, which was less than 60% prior to 1988, rose to approximately 90% from 1990 onwards and was associated with a near-elimination of pertussis reports, coincident with frequent fade-outs. Three consecutive regular epidemics in the years 1982, 1986 and 1990 are evident. We depict the square-rooted weekly incidence across the 32 boroughs in Fig 1B, which shows a comparable pattern of declining incidence throughout the 1990s and frequent fade outs until 2012. Thus, the epidemiology of pertussis over this time span is well described by periods of declining incidence (1982–1994), near absence of reports (1994–2012) and resurgence, culminating in the 2012 epidemic. This re-emergence occurred after the switch in 2004 from the whole-cell (DTwP) to the acellular (DTaP) vaccine in the routine schedule. This switch has been cited as one of the main potential explanations for the recent resurgence of pertussis in the UK [36, 76, 77]. We note, however, that most boroughs experienced a notable decline in vaccine coverage in the years leading to the vaccine switch (Fig 1A), with a drop in the mean vaccine coverage rates from 92% in 1997 to 82% in 2005. Finally, Fig 1C the annual incidence in boroughs is mapped (note square root scale).

**Fig 2:**
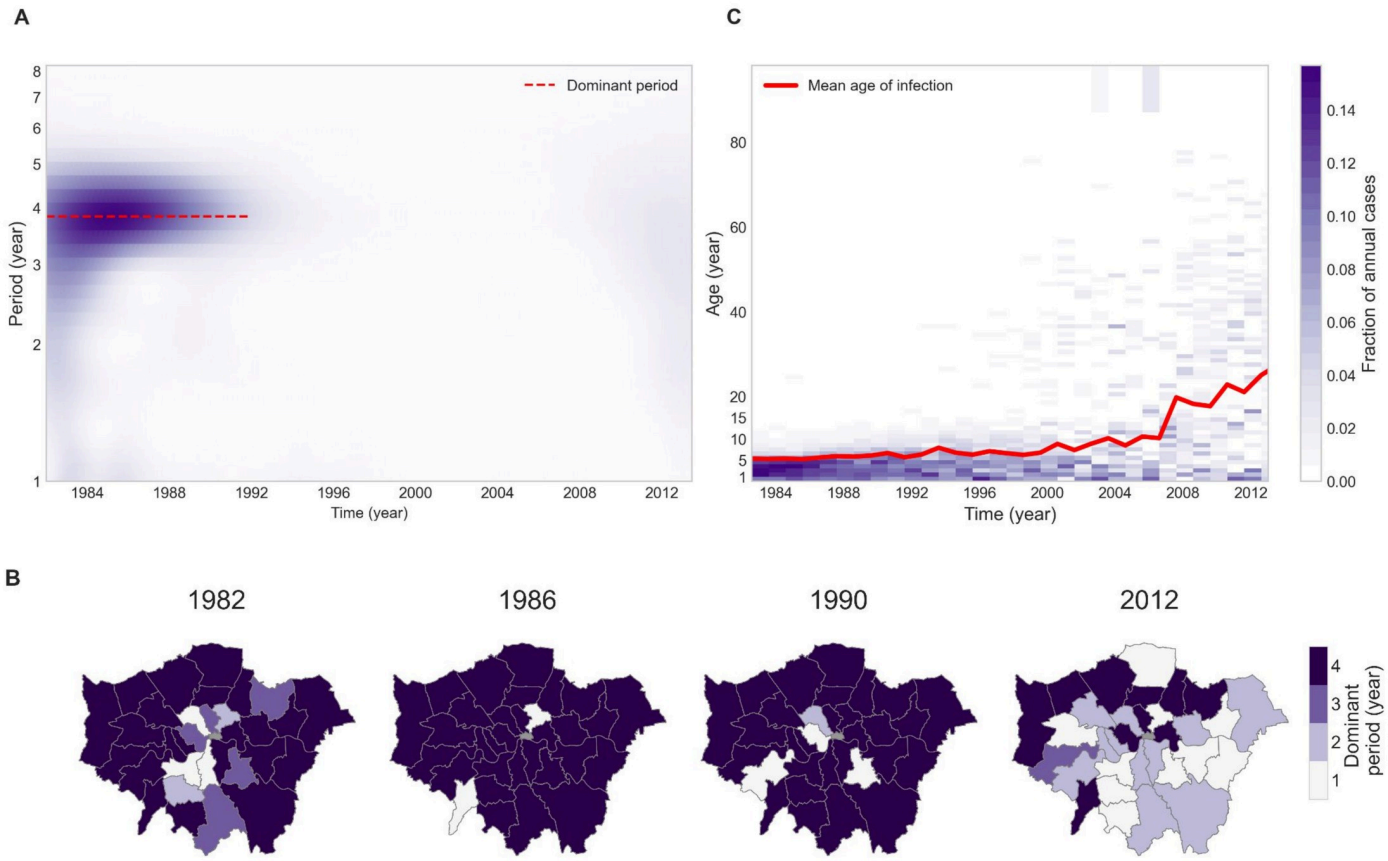
Changes in periodicity and age distribution of pertussis in Greater London and boroughs. (A) Local wavelet spectrum of cases time series of Greater London (B) Choropleth map of dominant period of the boroughs: In the first three epidemics (1982,1986,1990) most boroughs have dominant period of 3.5–4.5 years except a few with strong annual cycles. The epidemic cycles vanish by mid-1990s, and the 2012 resurgence does not exhibit as a periodic epidemic wave across the boroughs. Map base layers are obtained from London Datastore (https://data.london.gov.uk/dataset/statistical-gis-boundary-files-london) and are available from https://data.london.gov.uk/download/statistical-gis-boundary-files-london/9ba8c833-6370-4b11-abdc-314aa020d5e0/statistical-gis-boundaries-london.zip (The digital boundary file contains Office for National statistics data Crown copyright and database (2012) and contains Ordnance Survey data Crown copyright and database (2012)). (C) Annual number of pertussis in Greater London, discretized by age. The red line depicts mean age of infection by year.

The local wavelet power spectrum of aggregate data from Greater London is shown in Fig 2A and depicts a strong concentration of power within the 3.5–4.5 year band until the mid-1990s, after which no periodicity is apparent. A similar pattern is evident in local power spectrum of the boroughs (see S7 Fig). However, contrary to the trend in Greater London, fairly strong annual cycles are also observed in some of the boroughs (see regions shaded white in Fig 2A). We mapped the dominant period of the boroughs at the 1982, 1986 and 1990 epidemics in Fig 2B, which confirms that while most boroughs exhibit a strong quadriennial cycle, in some the annual component is stronger. Another notable observation in Fig 2B is the absence of any consistent periodicity across boroughs during the 2012 epidemic.

Increases in immunization coverage from late 1980s (Fig 1A) coincided with a notable shift in the age distribution of pertussis cases [45, 78, 79]. In Fig 2C, we depict the annual number of reported cases in Greater London discretized by age, illustrating that the burden of diseases is largely concentrated in 1–5 year-old children until 1988, after which it is restricted to very young infants and children aged 7 and older. Starting in the mid-1990s, a gradual increase in the proportion of adult cases is observed, followed by a sharp rise from 2006 onward. A similar trend has been previously reported for England & Wales [45], the US [80], and Canada [81]. This shift in the age distribution of cases to older age groups is reflected in the mean age of infection, which gradually increases from approximately 5 years in 1982 to ∼10 years by 2006; consistent with increasing national vaccine coverage rates over this period (Fig 1A). A striking observation from Fig 1C is the steep increase in the mean age of infection from 2006 onward, reaching 27-years old by 2013.

**Fig 3:**
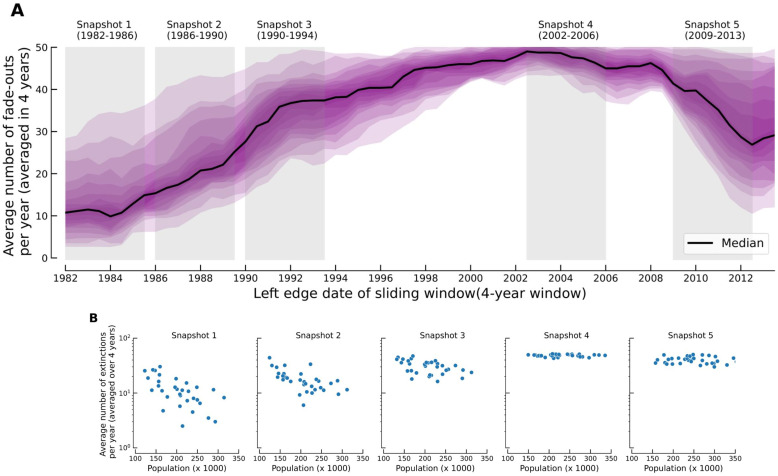
Fade-out profile of pertussis in Greater London and boroughs. (A) Distribution of number of fade-outs among boroughs of London. Shaded regions show the middle inter-percentile bands for different percentile values (inter-percentile range of 1% to 90%)(B) The number of fade-outs vs population size at four temporal snapshots (1982–1986, 1986–1990, 1990–1994, 2002–2006, and 2009–2013).

We also examined the fade-out frequency profile of pertussis over this period, as shown in Fig 3A. The black line shows the median number of fade-outs across Greater London and the purple shaded regions depict the middle inter-percentile bands for different percentile values. The figure illustrates that the number of fade-outs in all boroughs grows until ∼2002, which is consistent with the steady reduction in incidence shown in Fig 1A. However, the rate of growth in the number of fade-outs varies across boroughs. From 2002 onward, the number of fade-outs gradually declined until 2008, followed by a steep reduction leading up to the 2012 epidemic. Similar to 1980s, the rate of increase in the number of fade-outs during the resurgence varies significantly across boroughs. In Fig 3B, we depict the number of fade-outs at different time snapshots of Fig 3A as a function of the borough population size. In 1982, an exponential decline in the number of fade-outs with population size is evident. This relationship between fade-outs and population size becomes increasingly tenuous over time, ultimately disappearing in 2006.

**Fig 4:**
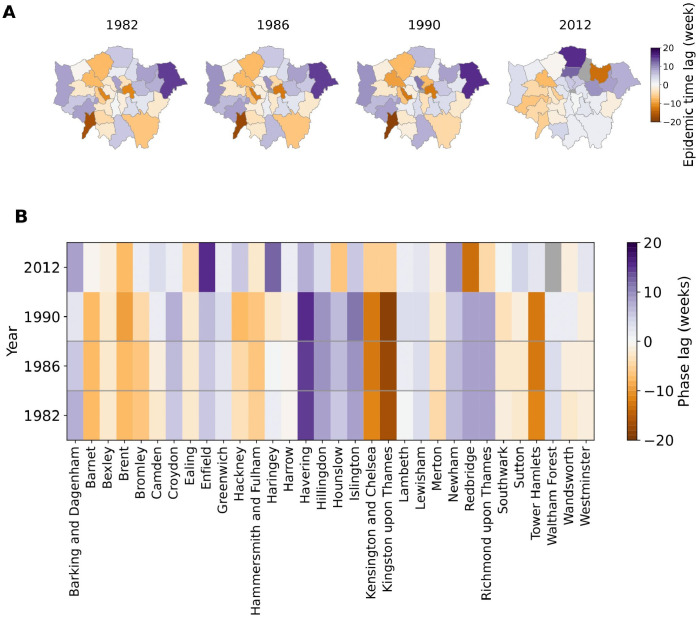
Pertussis epidemic phase lag among London boroughs in 1982, 1986, 1990 and 2012. Leading and lagging boroughs are shown in purple and orange respectively (A) Mapping of the phase lags at four different time snapshots. Map base layers are obtained from London Datastore (https://data.london.gov.uk/dataset/statistical-gis-boundary-files-london) and are available from https://data.london.gov.uk/download/statistical-gis-boundary-files-london/9ba8c833-6370-4b11-abdc-314aa020d5e0/statistical-gis-boundaries-london.zip (The digital boundary file contains Office for National statistics data Crown copyright and database (2012) and contains Ordnance Survey data Crown copyright and database (2012)). (B) Phase lag across time, depicting a consistent spatial organization among the boroughs at the first three snapshots.

Results of the epidemic time lag analysis are shown in Fig 4. The phase relationship among boroughs at the four epidemic snapshots is mapped in Fig 4A. Focusing on the early era (1982–1994), the most striking feature in this plot is the consistent spatial organization of boroughs across the three epidemics at 1982, 1986 and 1990. This is illustrated in Fig 4B, which shows the variation in borough phase lags across these three snapshots. Despite minor variations in relative phase lags in some boroughs, the spatial organization of the boroughs is consistent with each borough either consistently among the leaders of the epidemic wave, or lagging. The figure reveals four distinct foci (shaded purple) in the northeast, west, south, and the center of London that appear to lead the epidemic wave from 1982 to 1990. We found the spatial hierarchy of boroughs during the 2012 epidemic to be qualitatively different from the previous three snapshots. However, due to the sparsity of cases in some of the boroughs, the 2012 time lags must be interpreted with caution. The consistent spatial hierarchy of boroughs during the first three snapshots (1982, 1986 and 1990) prompted us to investigate the potential determinants of this spatial organization, by examining the statistical association between phase lag and demographic, socio-economic and geographical heterogeneity among boroughs.

### Determinants of epidemic phase lag among boroughs

Here we describe the statistical analysis of phase lags against various demographic, socio-economic and geographical factors. Due to the omission of household composition variables from the 2011 census and the switch from Socio-economic group (SEG) classification to The National Statistics Socio-economic classification (NS-SEC) [82], we used the first three snapshots (1982,1986,1990) to perform multivariate analysis and conducted univariate analysis on 2012 snapshot.

#### 1982–1990

The relative performance of the linear and FFNN regression models, as quantified by the coefficient of determination (*R*^2^), is presented in S8 Fig. As shown in S8(A) Fig, OLS fails to generalize well to the test set and thus is overfitting. The lasso and ridge models perform slightly better in this respect, however, none of them outperforms the FFNN model, shown in S8(B) Fig. The FFNN model performed relatively well on all three data sets (train, validation and test). Comparing the coefficients of determination between the three datasets indicates that the model is slightly overfitted to the train dataset.

**Fig 5:**
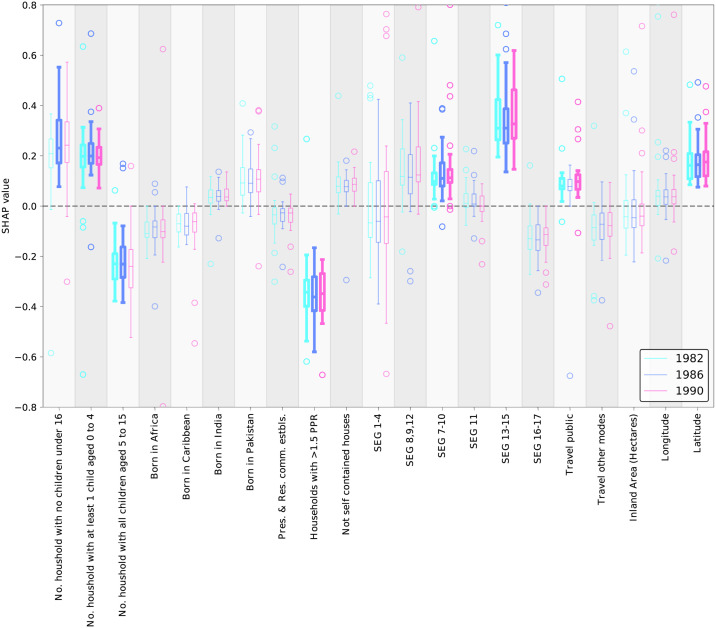
Aggregated SHAP values, representing the marginal contribution of each feature to predicted phase lags by the FFNN model. After fitting the FFNN model on the entire dataset, local SHAP values are obtained for each data point and aggregated over each time snapshot, distinguished with different colors. Values plotted in bold indicate statistical significance.

We used the trained FFNN model to make prediction for all the samples and estimated the local SHAP value for each sample to obtain a local feature importance for each feature at the corresponding sample. The local SHAP values quantify the linear association between each feature and the phase lag as the target variable at the corresponding sample point (see supplementary section 7 for more information on SHAP). After grouping the samples by year, we plotted the local SHAP values for each feature as shown in Fig 5. It is important to note that (i) a positive SHAP value indicates a positive association between that feature and a borough’s phase lag, and (ii) boroughs with a positive phase lag lead the epidemic wave.

Overall, we find four distinct characteristics of a borough affect their relative phase position in London: household composition, socio-economic factors, transportation traits and latitude (Fig 5). Taking these in turn, we detect a notable difference in SHAP values among the three features representing household composition, specifically the *Number of households with children aged 0–4* has a positive relationship with phase in all three outbreak years, indicating an earlier epidemic take-off. In contrast, *Number of households with children age 5–16* is negatively associated with phase lag in the 1982 and 1986 outbreaks. We note that the *Number of households with no children* is positively associated with phase lags in 1986. *Households with > 1.5 PPR* also shows strong negative association, which counter-intuitively suggests that boroughs with less densely-populated household lead the epidemic wave. We also found socio-economic drivers of epidemic phase, namely *SEG 13,15* (quantifying the number of farmers and agriculture workers in a borough), which stands out as the feature with strongest positive association with phase in all three years. A weaker positive association is also observed between *SEG 7–10* (Skilled manual workers) and phase lag. The remaining features to highlight are *Travel public* (number of people who use public transportation to travel to work) and *latitude* which exhibit a positive association with phase, though the relationship with public transportation is only for the 1982 and 1990 outbreaks.

**Fig 6:**
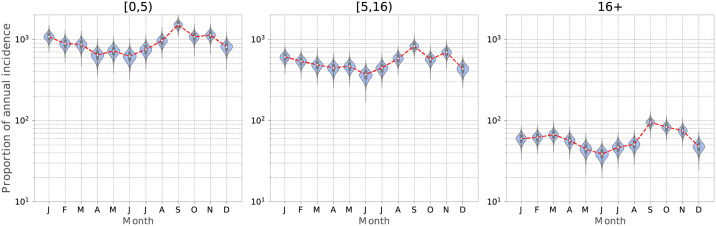
Age-specific seasonality in incidence (1982–1990). The figure demonstrates the year-to-year variation in the reported cases during each month for each age group. The distributions are generated from 10000 bootstrap samples of size 1000. The proportions for each age group is scaled by total cases in the corresponding age group. The relative incidence in each age group (scaled by the number of cases in the adult age group) is: 15.2, (11083 total cases), 8.88 (6474 total cases), and 1 (729 total cases), respectively. The amplitude of seasonality for each age group (calculated as (max-min)/average) is: 0.974, 0.847 and 0.926, respectively. Similarly, the standard deviation in intra-annual incidence for each age group is: 0.0228, 0.0196 and 0.0231.

To dissect the relationship between the age composition of households and phase, we analyzed the age-specific seasonality in reported cases for the focal epidemic years of 1982, 1986 and 1990. In Fig 6, we present the within-year variation in reported cases for each age group and find that incidence is 1.7-fold higher among young children (0–5 years old), compared with 5–16 year-olds. While the broad patterns of seasonality across age are comparable, with troughs in May-July and a peak in September, the overall amplitude of seasonal variation in incidence—calculated as (max-min)/mean—is higher for the youngest age group (0.974 Vs 0.847). We note that the amplitude of seasonal variation is similarly higher in the adult age group (0.926) than 5–15 year-olds. Taken together, these findings suggest that boroughs with many households composed of young children account for most intense transmission, experience the shallowest summer troughs and thus lead epidemics in London.

#### 2012

The results of univariate regression analysis of 2012 epidemic is shown is S3 Table. Among the census variables, we found a moderate negative correlation between epidemic timing and *Born in Pakistan* (*r*_*Pearson*_ = −0.4, *P* = 0.007), i.e. boroughs with a greater Pakistan-born population lag the epidemics. We did not find any significant association between other census variables and epidemic phase.

To examine the potential association between the decline in vaccine coverage rates and epidemic time lags, we used the annual birth and immunization data to calculate the cumulative number of unvaccinated children from 1997 to 2012 in each borough, and divided the total count by the population of each borough (as reported in census 2011) to find the *Ratio of cumulative unvaccinated children to total population*. As shown in S3 Table, we found a moderate positive correlation between this feature and phase lag (*r*_*Pearson*_ = 0.4, *P* = 0.019); that is, boroughs with greater ratio of unvaccinated children tend to lead the epidemic.

## Discussion and conclusion

In this paper, we have examined the spatio-temporal dynamics of pertussis incidence in the boroughs of Greater London from 1982 to 2013. The dynamics of pertussis over this period can be characterized by three stages: a period of declining trends with four-year inter-epidemic cycles from 1982 to 1994, a deep trough until 2006, and the re-emergence of the disease leading to an outbreak in 2012. There was an increase in the estimated immunization coverage in Greater London from 55–60% in the early to mid-1980s to approximately 90% by the early 1990s (Fig 1A). Our study indicates that coincident with increasing vaccine coverage until mid-1990s there were predictable systematic trends in pertussis epidemiology, namely an overall decline in pertussis incidence (Fig 1A), smaller epidemic sizes (Fig 2A), a shift in the age distribution of cases towards older individuals (Fig 2B) and a rise in the frequency of fade-outs (Fig 3). The subsequent re-emergence stage is characterized by a steep decline in fade-out frequency (Fig 3) and a sharp increase in the mean age of infection (Fig 2B), however, we did not detect any periodicity in the wavelet spectrum of reported incidence (Fig 2A).

Various potential explanations have been offered in the literature for the recent re-emergence of pertussis in highly vaccinated populations such as England and Wales, including changes in the vaccine schedule and diagnostics [32], vaccine failure [36, 37, 83–87], waning of vaccine immunity [37, 85, 88–90], differences in the efficacy of acellular and whole cell vaccines [36, 37, 86], changes in the population contact structure [36, 45], the impact of asymptomatic transmission [89, 91–94] perhaps associated with the introduction of acellular vaccines [37, 77], and failure in the degree of vaccine protection [32, 83, 95], possibly arising from vaccine-driven evolution [96, 97]. In addition, it is worth pointing out that there was a notable decreasing trend in the vaccine coverage rates across the boroughs of London in the years preceding the 2012 epidemic (Fig 1A), whose potential role in the re-emergence of pertussis requires further systematic investigation.

Despite the pronounced trends in pertussis incidence from 1982 to 1990, the relative ranking of the timing of outbreaks in boroughs, quantified through their relative phases (Fig 4A), did not exhibit substantial variation and instead revealed a reasonably consistent pattern of spatial organization (Fig 4B). To understand the mechanisms that may underpin the observed geographic motif, we assessed the association between borough-specific phases and a range of demographic, socio-economic and geographical factors using a combination of approaches, including linear regression and neural networks.

Linear regression models have been extensively used in the literature to investigate the drivers of epidemic asynchrony for various infectious disease systems [1, 3, 5]. These models are easy to implement and are inherently interpretable—that is, the relative importance of each variable for prediction is transparently quantified via the estimated coefficients. However, this convenience in modeling and interpretation comes at the expense of accuracy as these models, by definition, are incapable of capturing potentially nonlinear relationships between predictor variables and the target variable (S8 Fig). On the other hand, more complex models may provide improved prediction accuracy, but their results might not be readily interpretable. To overcome this hurdle, we adopted a novel methodology that has allowed consideration of possible nonlinearities in the model, while yielding straightforward interpretability between underlying demographic, socio-economic and geographical factors and the spatial organization of London boroughs. We have taken advantage of SHAP-based feature importance to quantify the relative contribution of each factor to model predictions. Our initial univariate regression analysis identified an association between 11 of the predictor variables and relative phase lags (*P* < 0.05) (See S1 Table and S9–S15 Figs). However, the subsequent SHAP-based analysis indicated that only a small subset of features contributed to predictive performance. This difference was not surprising given the high degree of correlation among the variables (as shown in S5 Fig).

The important features during the 1982–1990 epidemics, identified via SHAP analysis, were the age composition of households, agricultural workers, skilled manual workers, latitude, population of public transport commuters and high-occupancy households as key predictors of borough phase. We found the differences in age-specific seasonality of incidence as a key determinant of epidemic phase lag among the boroughs during this period. An earlier epidemic peak in September in children 0–5 years of age and adults causes the boroughs with higher population of these age groups to lead the epidemic wave. A second peak in November in adolescents can explain the epidemic lag in boroughs with more households with children 5–15 years old of age. A similar pattern in age-specific seasonality has been reported in a past study in Massachusetts [98, 99] and in the US overall [100]. During the 2012 epidemic, our univariate regression analysis indicated the size of Pakistan-born population to be negatively correlated with timing of epidemic. This may be indicative of some underlying correlation between infant immunization coverage and these socio-economic characteristics.

As mentioned previously, prior work leads us to suspect the outbreak phase of a borough to be impacted in part by susceptible recruitment, which in turn is affected by vaccine uptake [3]. However, we have been unable to obtain vaccine coverage at the borough level for the 1982–1990 period, with 1997 the first year for which these data can be obtained. Therefore, we examined the association between the vaccine coverage and epidemic phase in 1994 (borough-level incidence data post-1994 were too sparse to quantify phase lags). As shown in S17 Fig, no significant association was observed between the two. On the other hand, we found a moderate positive association between *Ratio of cumulative unvaccinated children to total population*, a proxy for susceptible pool size, and the timing of 2012 epidemic across the boroughs.

Our study paints a different picture of the spatial ecology of an infectious disease at fine spatial scale. Past work on traveling waves of measles in England and Wales [1] uncovered a *source-sink* relationship between large cities serving as the source, leading biennial outbreaks and their satellite towns and villages acting as the sink, with lagging epidemics. In contrast, our study shows that at the borough level, epidemic foci located in the northeast, west, south, and center of London lead the 4-year epidemic wave of 1982–1990. In another study of the timing of seasonal rotavirus outbreaks across the US, it was shown that state-specific birth rates can explain epidemic timing [3]. We found incidence in infants and children 0–4 years of age as a determinant in timing of the epidemics spanning 1982–1990. Finally, a number of previous studies have argued for a *pacemaker mechanism* in driving epidemic cycles [5, 101–103], where a group of dynamically synchronous foci act as local rhythm setters. The identification of the aforementioned foci in the northeast, west, south, and center of London would be consistent with this explanation. Understanding the mechanisms that determine which populations assume the role of pacemaker remains an important area of future research.

More broadly, we report a transition from spatially organized waves in the decline phase of this period to a largely unstructured mosaic in the resurgence era. This observation is consistent with a previous study of spatial synchrony in pertussis epidemics in US states, where organized waves in the wP era gave way to spatially disorganized contemporary epidemics [5]. It is tempting to speculate regarding whether the switch to acellular vaccines for the routine schedule in 2004 [41] played a part in these transitions, especially given the concern over increased likelihood of asymptomatic infections in aP vaccinees [37, 77, 91]. This is an interesting possibility that warrants careful consideration as we strive to understand the factors that may have played a role in the transformation of the spatial epidemiology of pertussis in these populations in recent decades.

## Supporting information

S1 Fig Interpolation of demographic and socioeconomic variables from decennial censuses. (PDF)

S2 Fig Variation of demographic and socioeconomic variables across boroughs of London—1982. (PDF)

S3 Fig Variation of demographic and socioeconomic variables across boroughs of London—1986. (PDF)

S4 Fig Variation of demographic and socioeconomic variables across boroughs of London—1990. (PDF)

S5 Fig Correlation matrix of geographic and census variables. (PDF)

S6 Fig Preprocessing and transformation steps of case count time series to obtain phase lags. (PDF)

S7 Fig Evolution of pertussis incidence in the boroughs of Greater London exhibited in the frequency domain. (PDF)

S8 Fig Comparison of true phase lag vs predicted phase lag by different models. (PDF)

S9 Fig Univariate linear regression coefficients. (PDF)

S10 Fig Univariate regression, bivariate Gaussian kernel density contours 1. (PDF)

S11 Fig Univariate regression, bivariate Gaussian kernel density contours 2. (PDF)

S12 Fig Univariate regression, bivariate Gaussian kernel density contours 3. (PDF)

S13 Fig Univariate regression, bivariate Gaussian kernel density contours 4. (PDF)

S14 Fig Univariate regression, bivariate Gaussian kernel density contours 5. (PDF)

S15 Fig Univariate regression, bivariate Gaussian kernel density contours 6. (PDF)

S16 Fig Multivariate linear regression coefficients. (PDF)

S17 Fig Ranking of the London boroughs by phase lag at 1994 vs their ranking by the vaccine coverage in 1997 (Immunization rate at the first birthday). (PDF)

S1 Table Results of univariate regression between epidemic phase lag of London boroughs and census features, 1982–1990. (PDF)

S2 Table Results of multivariate regression between epidemic phase lag of London boroughs and census features, 1982–1990. (PDF)

S3 Table Results of univariate regression between epidemic phase lag of London boroughs and census features, 2012. (PDF)

S1 Text SHAP values. (PDF)
