# Supplementary material for: Dissecting recurrent waves of pertussis across the boroughs of London
Source: PLoS Comput Biol. 2022 Apr 14;18(4):e1009898. doi: 10.1371/journal.pcbi.1009898 (PMC9041754; doi:10.1371/journal.pcbi.1009898)
Supplement: S1 Fig — (PDF) [file pcbi.1009898.s001.pdf]

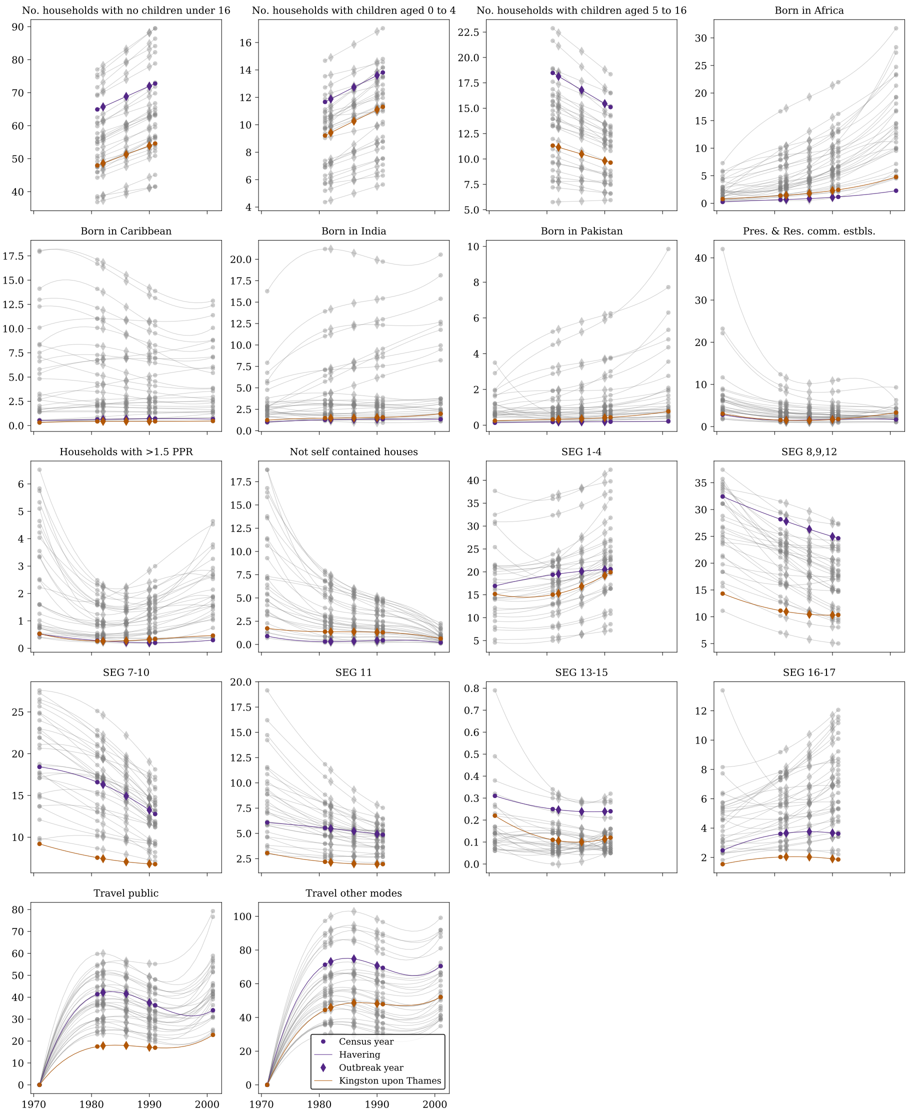

S1 Fig: Interpolation of demographic and socioeconomic variables from decennial censuses. Census variables are interpolated using B-splines to estimate the values in-between census years (census in the UK takes place every 10 years).
