# Supplementary material for: Dissecting recurrent waves of pertussis across the boroughs of London
Source: PLoS Comput Biol. 2022 Apr 14;18(4):e1009898. doi: 10.1371/journal.pcbi.1009898 (PMC9041754; doi:10.1371/journal.pcbi.1009898)
Supplement: S3 Fig — (PDF) [file pcbi.1009898.s003.pdf]

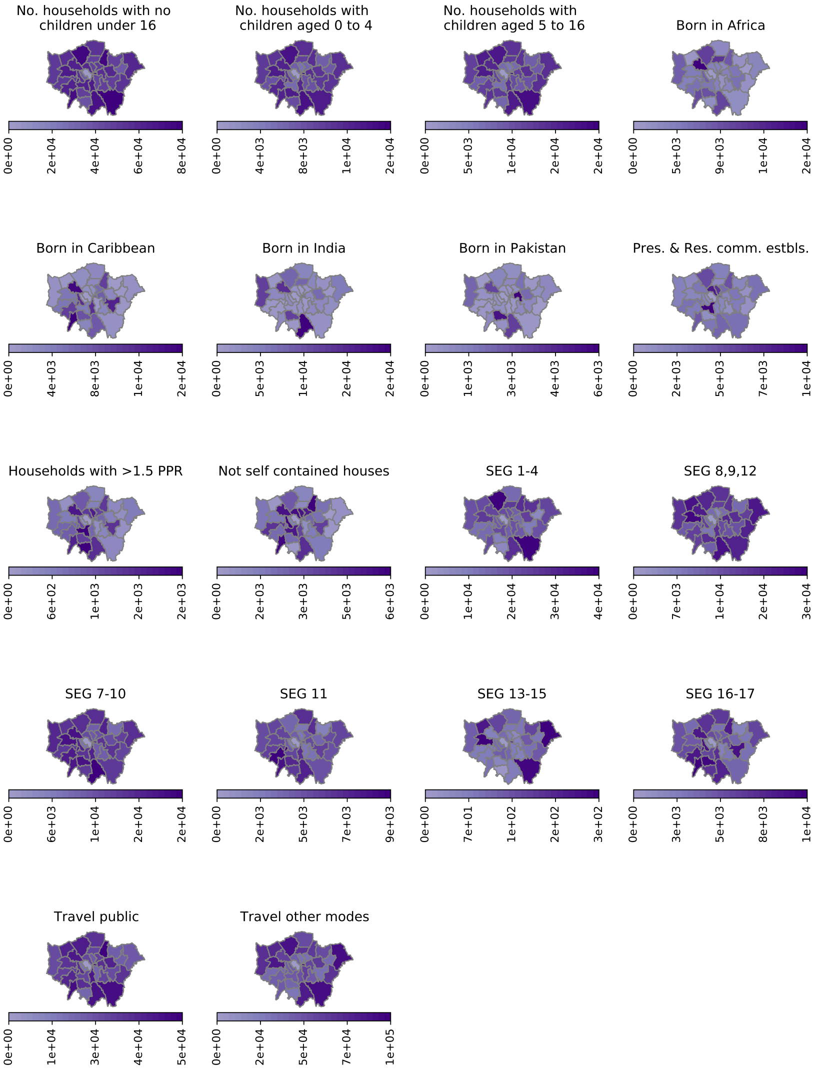

S3 Fig: Variation of demographic and socioeconomic variables across boroughs of London - 1986. Map base layers are obtained from London Datastore (https://data.london.gov.uk/dataset/statistical-gis-boundary-files-london) and are available from https://data.london.gov.uk/download/statistical-gis-boundary-files-london/9ba8c833-6370-4b11-abdc-314aa020d5e0/statistical-gis-boundaries-london.zip (The digital boundary file contains Office for National statistics data Crown copyright and database (2012) and contains Ordnance Survey data Crown copyright and database (2012)).
