# Supplementary figures and images for: Dissecting recurrent waves of pertussis across the boroughs of London
Source: PLoS Comput Biol. 2022 Apr 14;18(4):e1009898. doi: 10.1371/journal.pcbi.1009898 (PMC9041754; doi:10.1371/journal.pcbi.1009898)

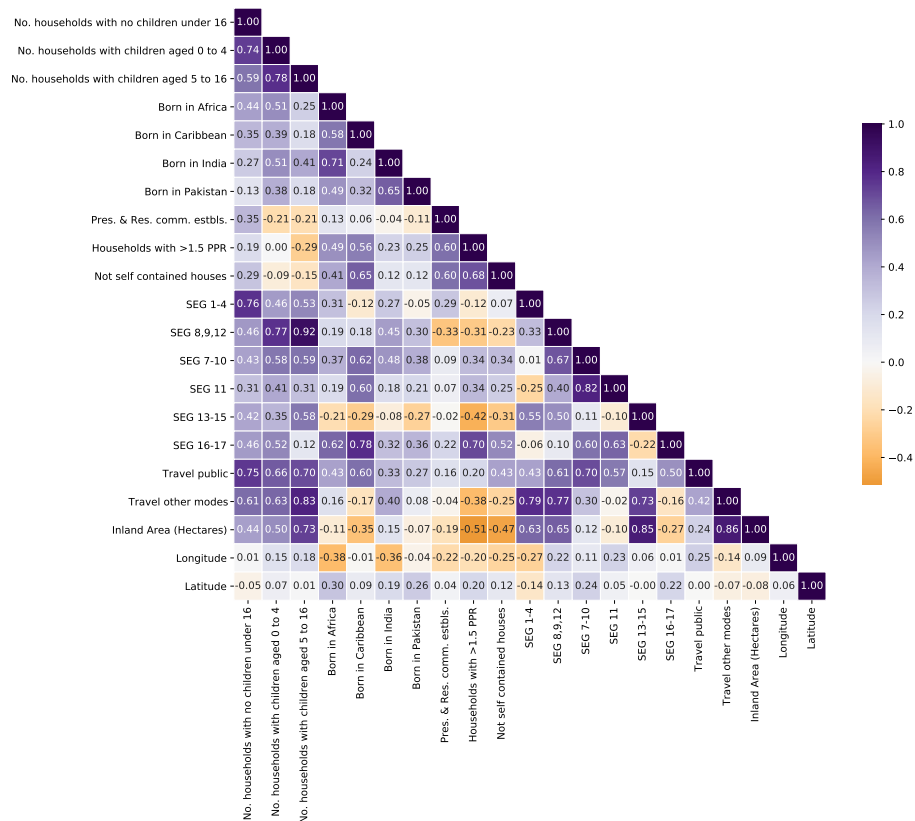

S5 Fig: Correlation matrix of geographic and census variables

Supplement: S5 Fig — (PDF) [file pcbi.1009898.s005.pdf]

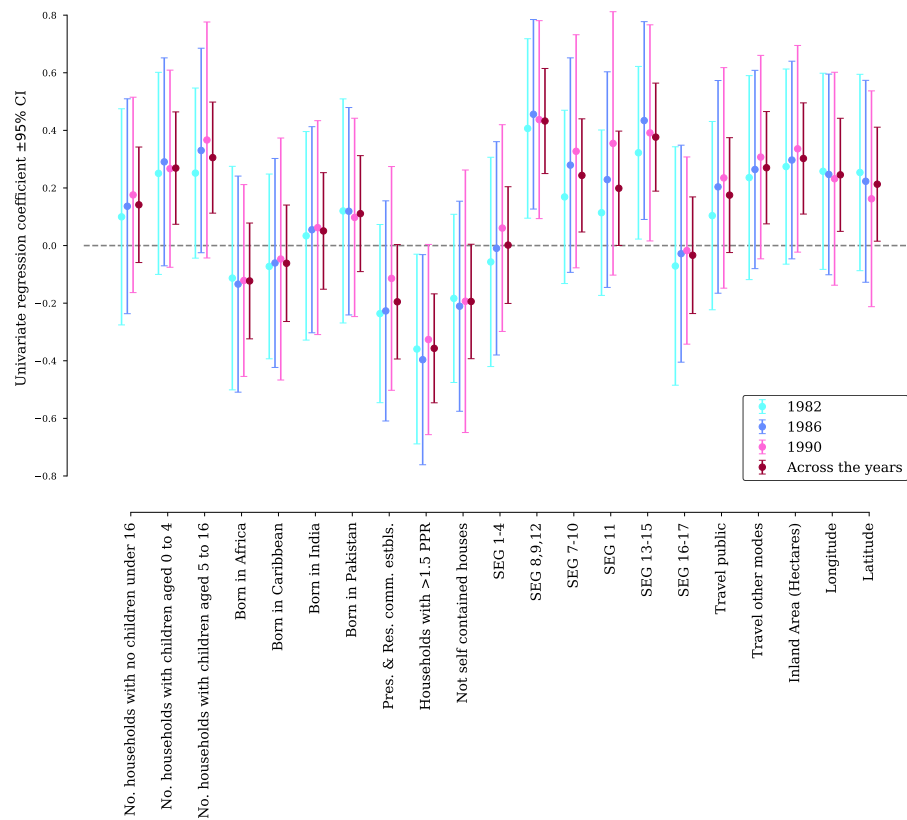

S9 Fig: Univariate linear regression coefficients

Supplement: S9 Fig — (PDF) [file pcbi.1009898.s009.pdf]

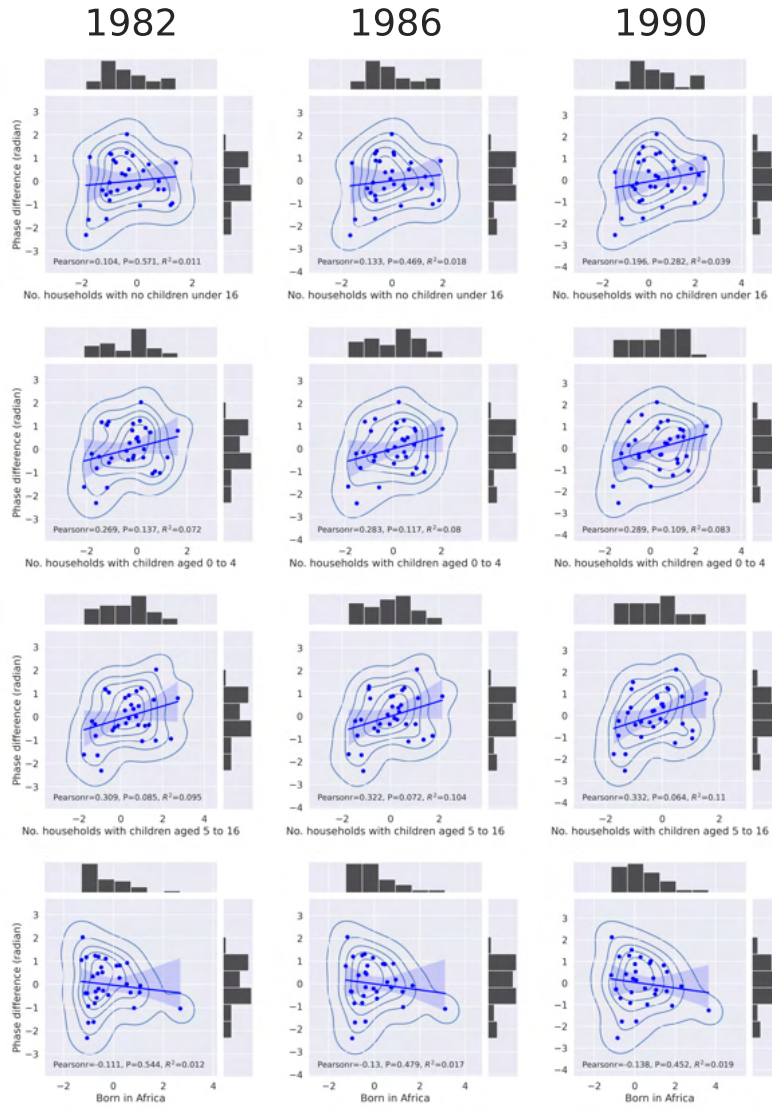

S10 Fig: Univariate regression, bivariate Gaussian kernel density contours

Supplement: S10 Fig — (PDF) [file pcbi.1009898.s010.pdf]

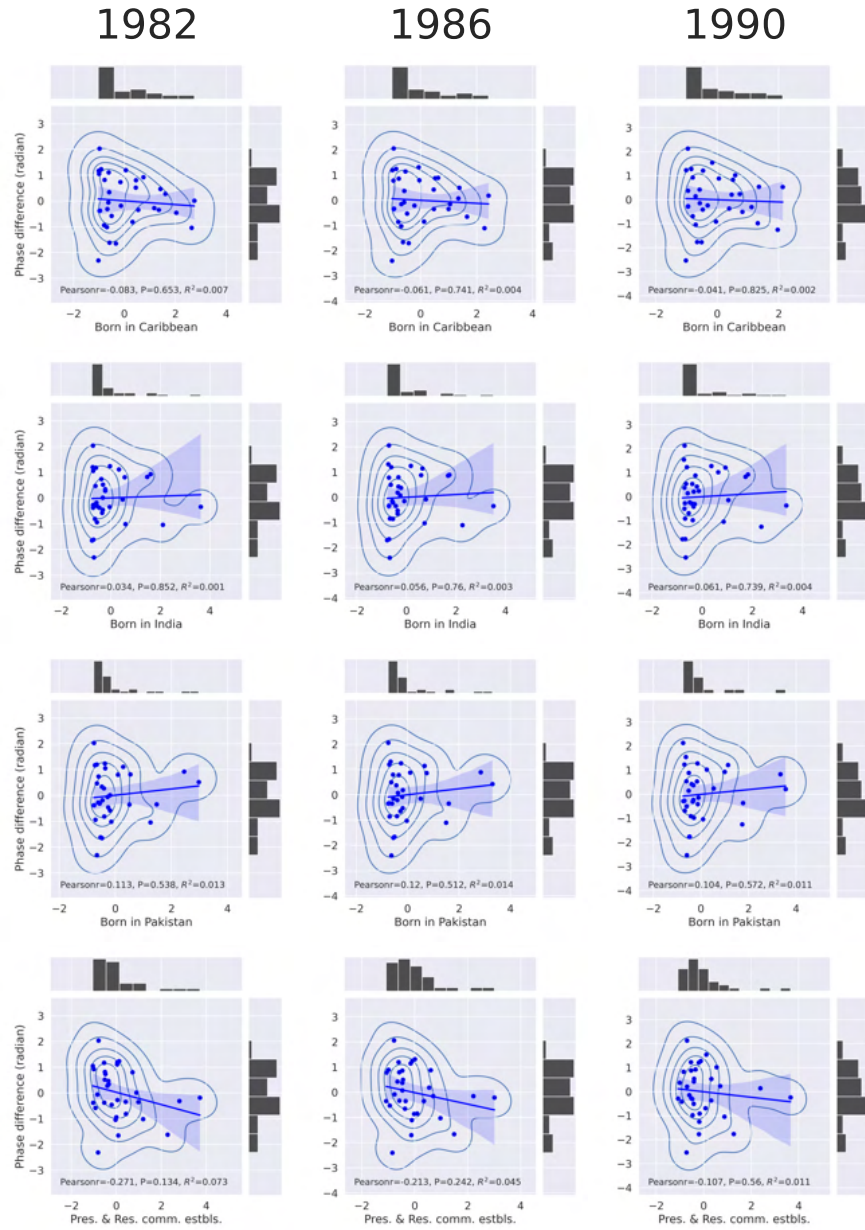

S11 Fig: Univariate regression, bivariate Gaussian kernel density contours

Supplement: S11 Fig — (PDF) [file pcbi.1009898.s011.pdf]

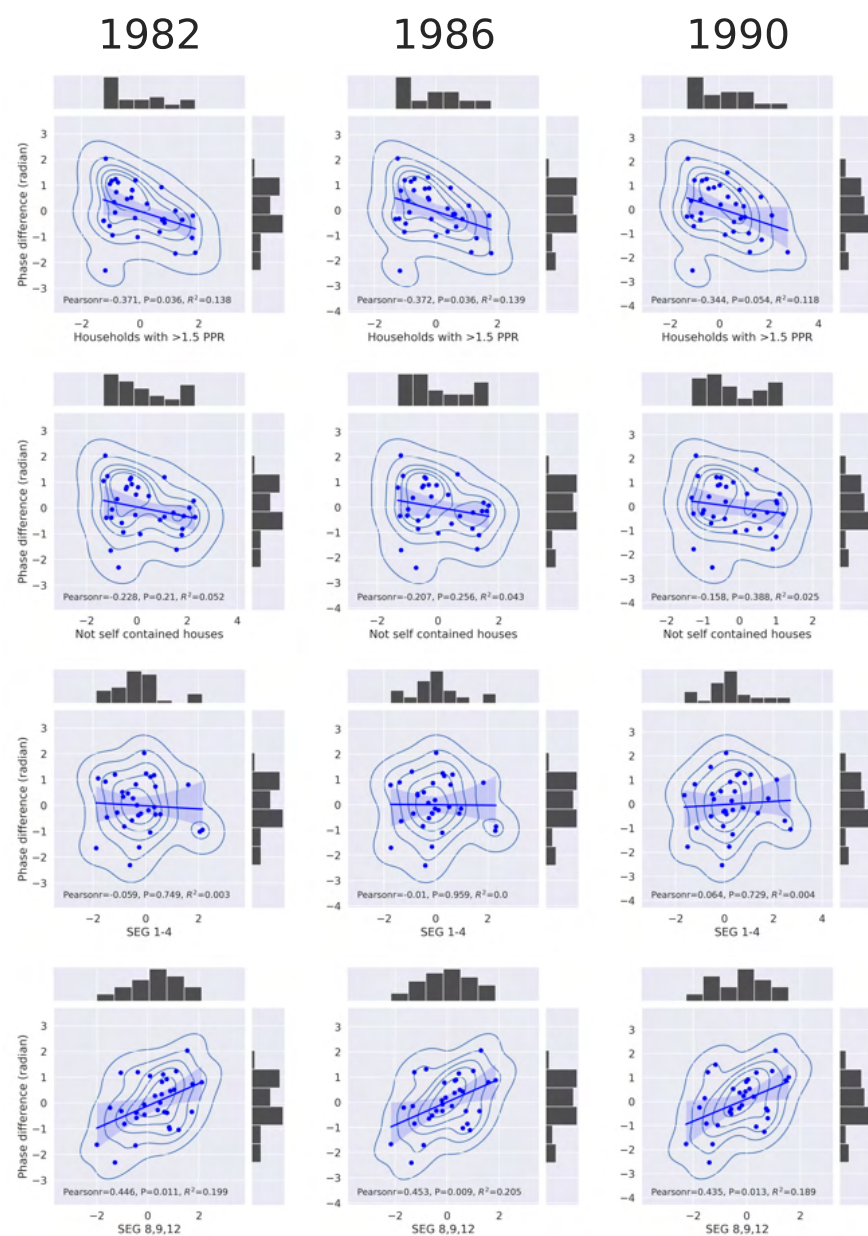

S12 Fig: Univariate regression, bivariate Gaussian kernel density contours

Supplement: S12 Fig — (PDF) [file pcbi.1009898.s012.pdf]

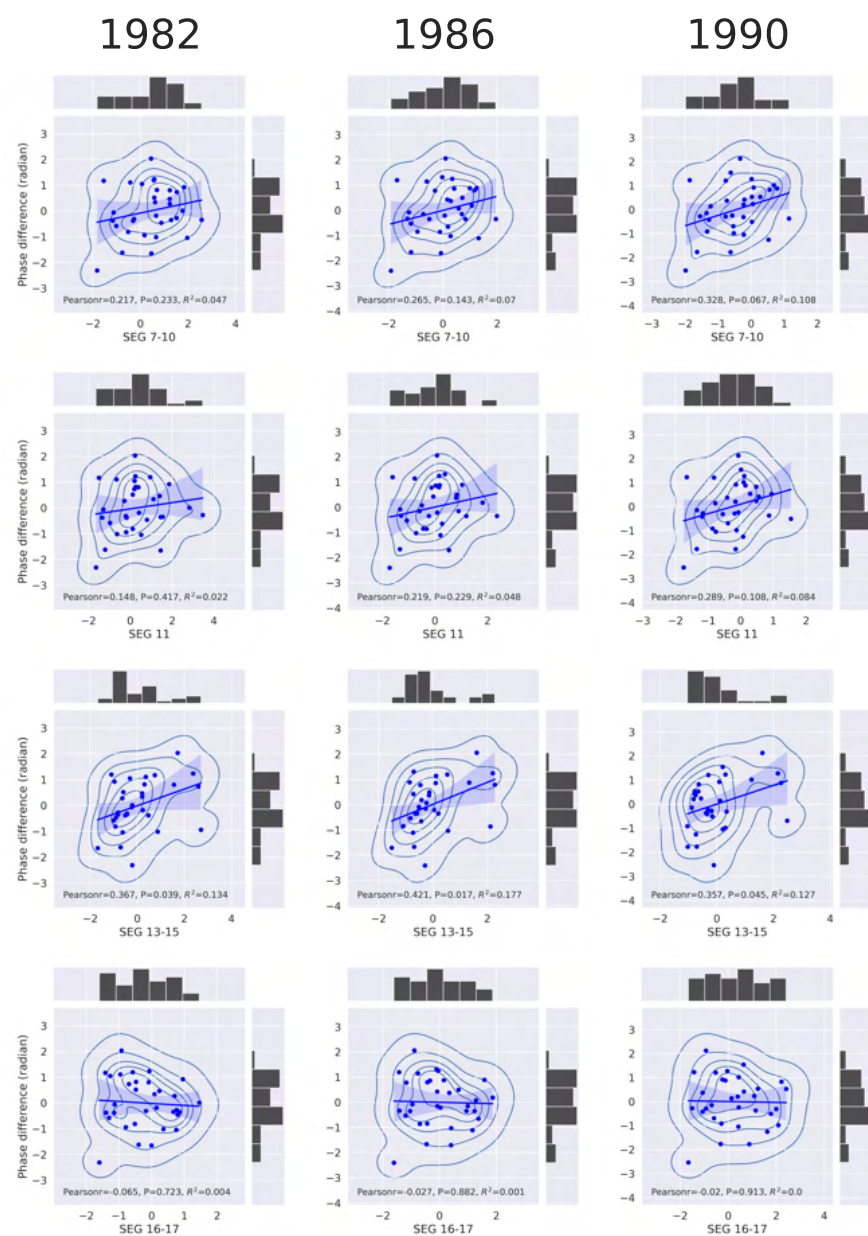

S13 Fig: Univariate regression, bivariate Gaussian kernel density contours

Supplement: S13 Fig — (PDF) [file pcbi.1009898.s013.pdf]

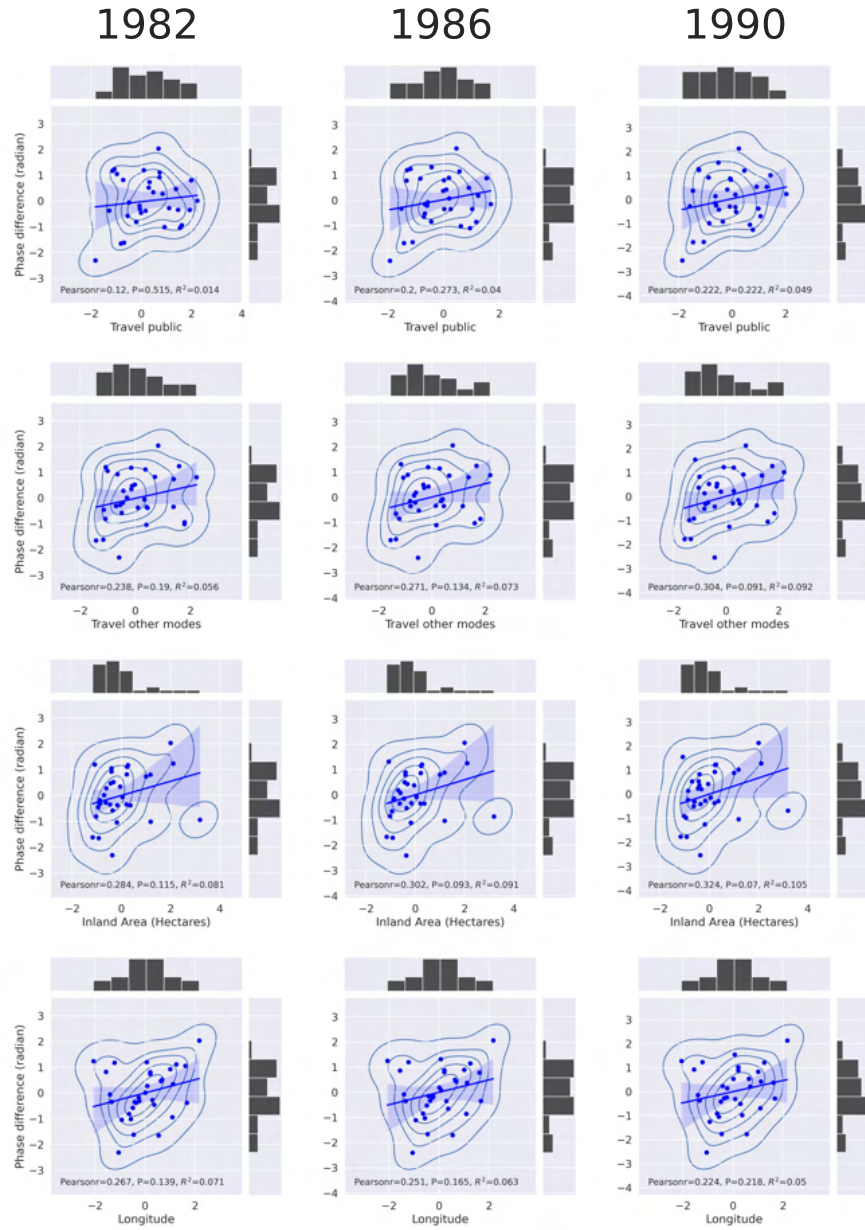

S14 Fig: Univariate regression, bivariate Gaussian kernel density contours

Supplement: S14 Fig — (PDF) [file pcbi.1009898.s014.pdf]

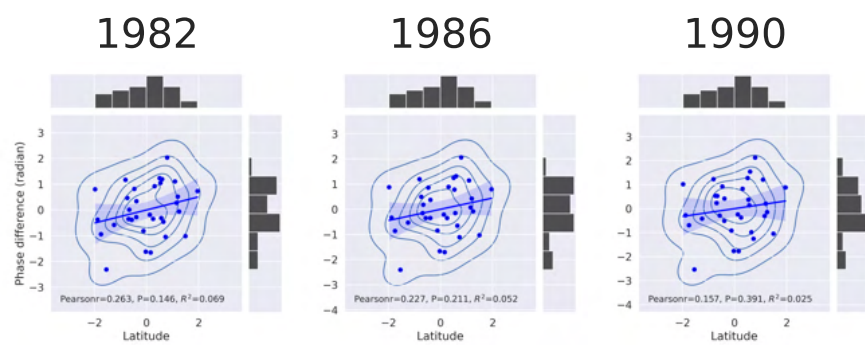

S15 Fig: Univariate regression, bivariate Gaussian kernel density contours

Supplement: S15 Fig — (PDF) [file pcbi.1009898.s015.pdf]

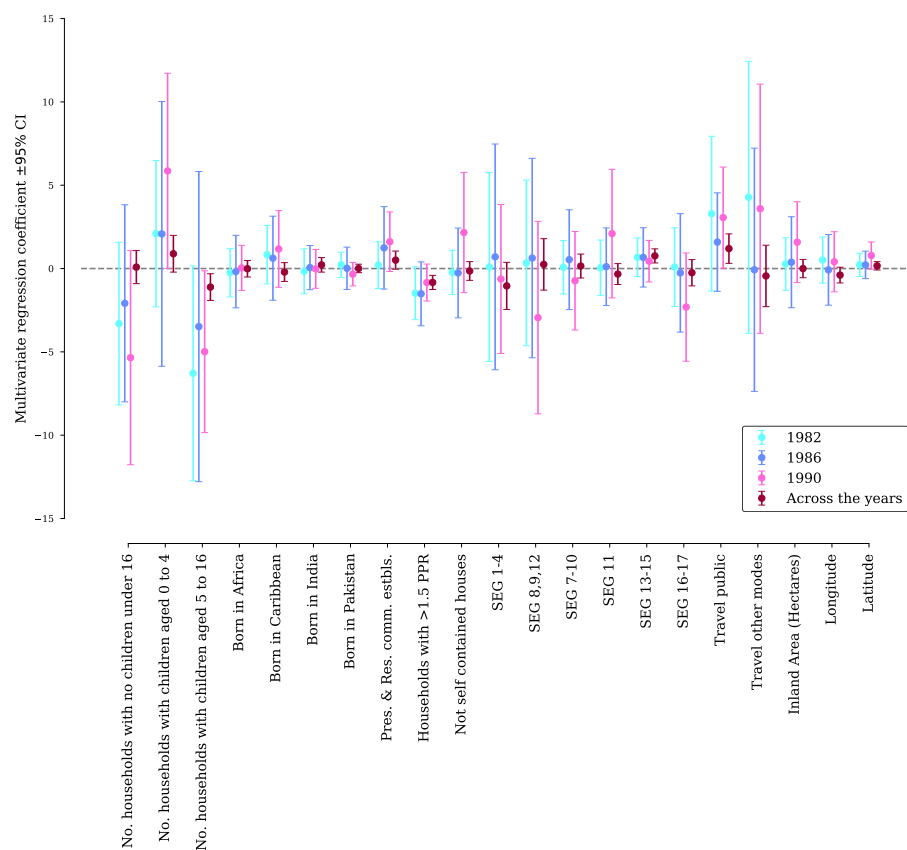

S16 Fig: Multivariate linear regression coefficients

Supplement: S16 Fig — (PDF) [file pcbi.1009898.s016.pdf]
