# Supplementary material for: Dissecting recurrent waves of pertussis across the boroughs of London
Source: PLoS Comput Biol. 2022 Apr 14;18(4):e1009898. doi: 10.1371/journal.pcbi.1009898 (PMC9041754; doi:10.1371/journal.pcbi.1009898)
Supplement: S6 Fig — (PDF) [file pcbi.1009898.s006.pdf]

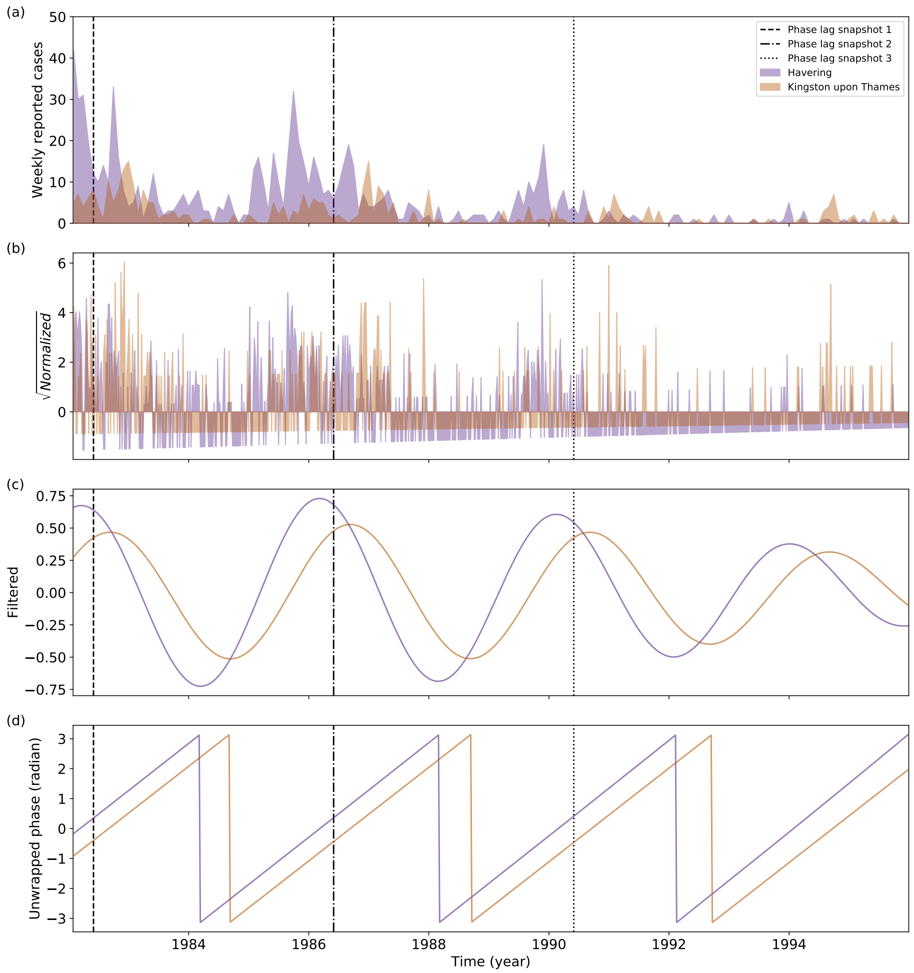

S6 Fig: Preprocessing and transformation steps of case count time series to obtain phase lags. For illustrative purposes, only results for most leading (Havering) and most lagging (Kingston upon Thames) boroughs of the 1982 epidemic are shown. (a) Case count time series (b) Squared rooted and normalized time series with mean zero and unit variance (c) Filtered within the period range of 3.5-4.5 years (d) Unwrapped phase values.
