# Supplementary material for: Dissecting recurrent waves of pertussis across the boroughs of London
Source: PLoS Comput Biol. 2022 Apr 14;18(4):e1009898. doi: 10.1371/journal.pcbi.1009898 (PMC9041754; doi:10.1371/journal.pcbi.1009898)
Supplement: S7 Fig — (PDF) [file pcbi.1009898.s007.pdf]

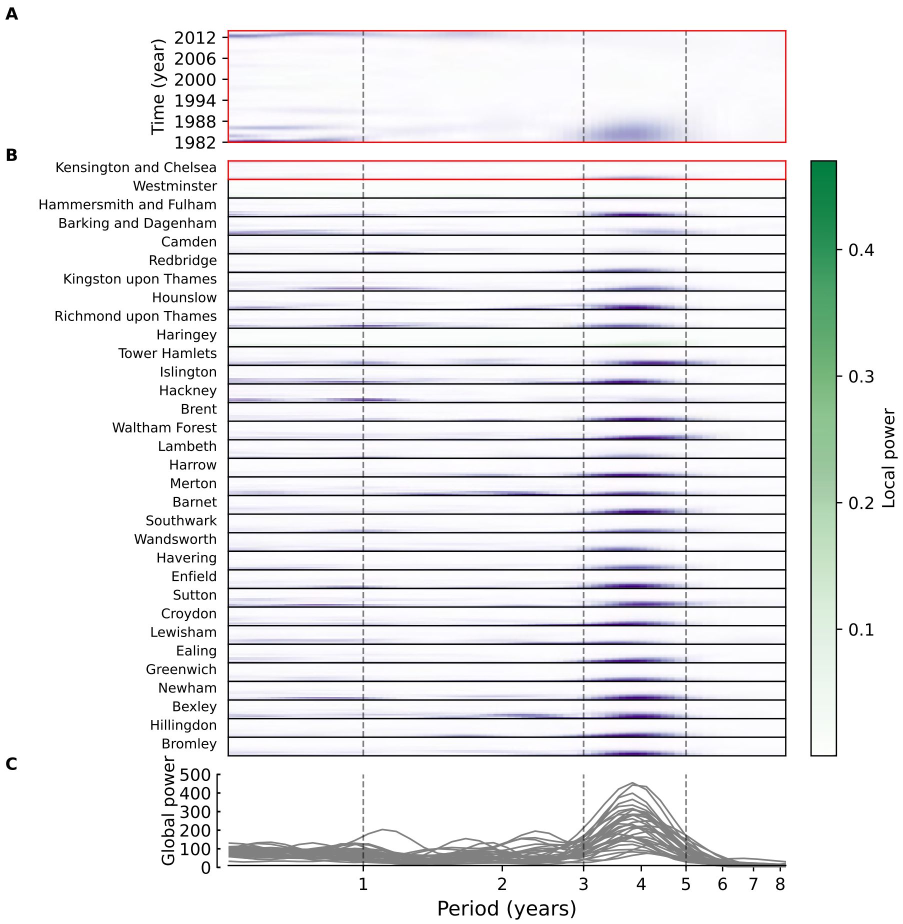

S7 Fig: Evolution of pertussis incidence in the boroughs of Greater London exhibited in the frequency domain (A) Local power spectra of Kensington and Chelsea shown for illustrative purposes (B) Local power spectra of boroughs obtained via continuous wavelet analysis (C) Global power spectra of boroughs
