# Supplementary material for: Dissecting recurrent waves of pertussis across the boroughs of London
Source: PLoS Comput Biol. 2022 Apr 14;18(4):e1009898. doi: 10.1371/journal.pcbi.1009898 (PMC9041754; doi:10.1371/journal.pcbi.1009898)
Supplement: S8 Fig — (PDF) [file pcbi.1009898.s008.pdf]

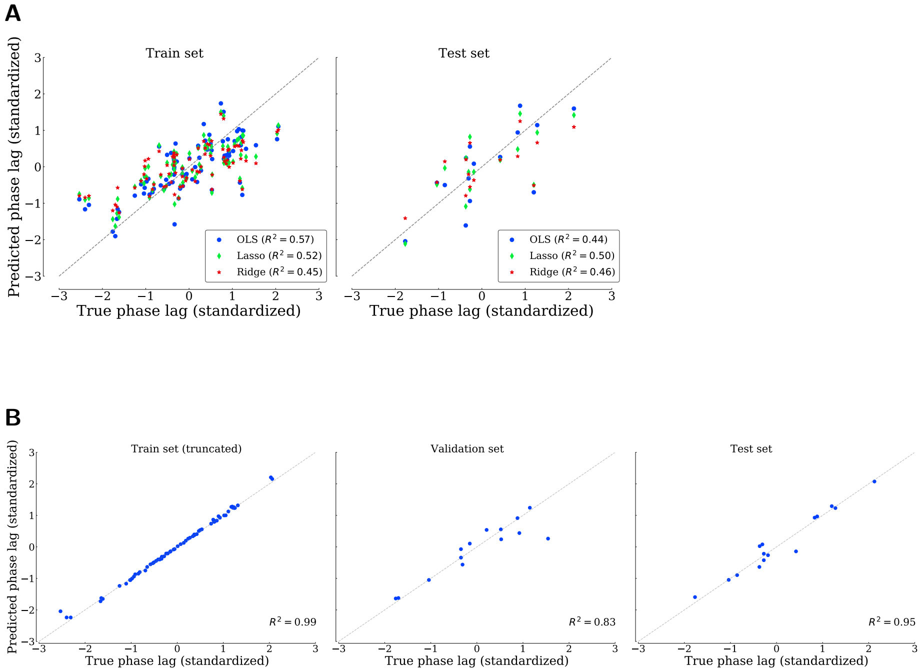

S8 Fig: Comparison of true phase lag vs predicted phase lag by the model (A) Ordinary least squared, lasso and ridge regression models (B) FFNN model.
