# Supplementary material for: Dissecting recurrent waves of pertussis across the boroughs of London
Source: PLoS Comput Biol. 2022 Apr 14;18(4):e1009898. doi: 10.1371/journal.pcbi.1009898 (PMC9041754; doi:10.1371/journal.pcbi.1009898)
Supplement: S17 Fig — (PDF) [file pcbi.1009898.s017.pdf]

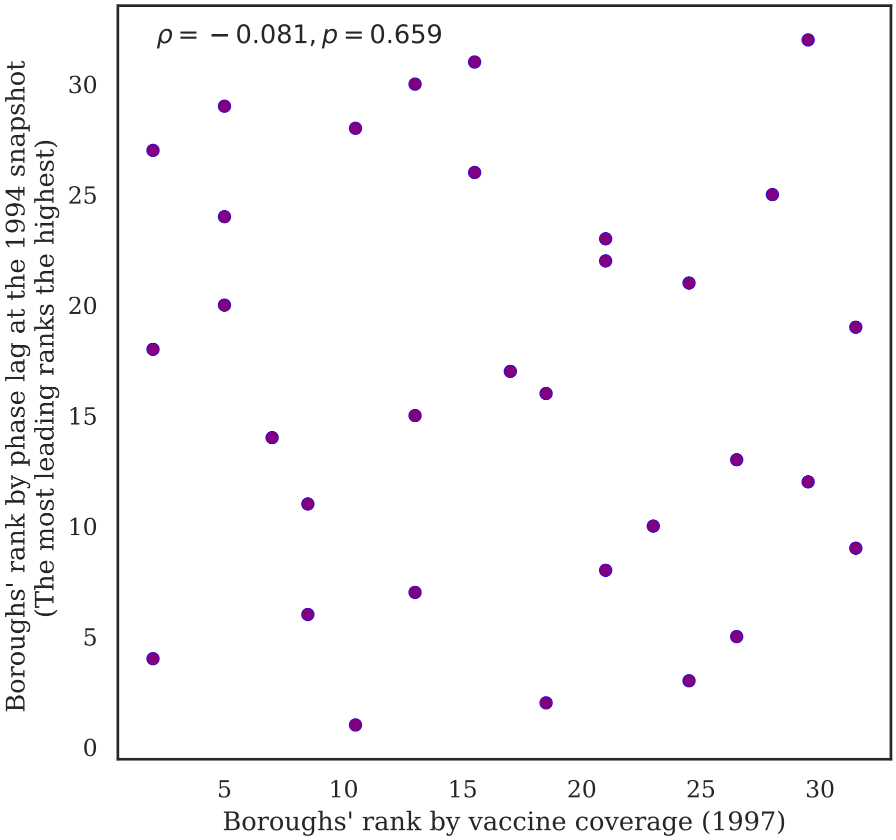

S17 Fig: Ranking of the London boroughs by phase lag at 1994 vs their ranking by the vaccine coverage in 1997 (Immunization rate at the first birthday).
