# Supplementary material for: Dissecting recurrent waves of pertussis across the boroughs of London
Source: PLoS Comput Biol. 2022 Apr 14;18(4):e1009898. doi: 10.1371/journal.pcbi.1009898 (PMC9041754; doi:10.1371/journal.pcbi.1009898)
Supplement: S1 Table — (PDF) [file pcbi.1009898.s018.pdf]

Features	*Pearson's r* (*P*-value) 1982	1986	1990	Across years
No. households with No children under 16	0.10(0.591)	0.13(0.461)	0.19(0.298)	0.14(0.164)
No. households with children aged 0 to 4	0.26(0.155)	0.28(0.110)	0.28(0.121)	**0.27(0.007)**
No. households with children aged 5 to 16	0.30(0.092)	0.32(0.067)	0.33(0.077)	**0.30(0.002)**
Born in Africa	-0.1(0.557)	-0.1(0.472)	-0.1(0.463)	-0.1(0.228)
Born in Caribbean	-0.0(0.648)	-0.0(0.736)	-0.0(0.822)	-0.0(0.547)
Born in India	0.03(0.850)	0.05(0.756)	0.06(0.734)	0.05(0.619)
Born in Pakistan	0.11(0.533)	0.12(0.504)	0.10(0.566)	0.11(0.277)
Pres. & Res. comm. estbls.	-0.2(0.129)	-0.2(0.235)	-0.1(0.553)	-0.1(0.054)
Households with > 1.5 PPR	**-0.3(0.033)**	**-0.3(0.034)**	-0.3(0.052)	**-0.3(0.000)**
Not self contained houses	-0.2(0.209)	-0.2(0.247)	-0.1(0.393)	-0.1(0.055)
SEG 1-4	-0.0(0.752)	-0.0(0.958)	0.06(0.732)	0.00(0.987)
SEG 8,9,12	**0.44(0.012)**	**0.45(0.008)**	**0.43(0.014)**	**0.43(0.0)**
SEG 7-10	0.21(0.260)	0.26(0.136)	0.32(0.109)	**0.24(0.015)**
SEG 11	0.14(0.423)	0.21(0.221)	0.28(0.123)	**0.2(0.049)**
SEG 13-15	**0.36(0.036)**	**0.42(0.014)**	**0.35(0.041)**	**0.37(0.000)**
SEG 16-17	-0.0(0.729)	-0.0(0.879)	-0.0(0.914)	-0.0(0.742)
Travel public	0.11(0.521)	0.19(0.268)	0.22(0.220)	0.17(0.084)
Travel other modes	0.23(0.184)	0.27(0.127)	0.30(0.085)	**0.27(0.007)**
Inland Area (Hectares)	0.28(0.108)	0.30(0.087)	0.32(0.065)	**0.30(0.002)**
Longitude	0.26(0.132)	0.25(0.158)	0.22(0.21)	**0.24(0.014)**
Latitude	0.26(0.139)	0.22(0.203)	0.15(0.383)	**0.21(0.035)**

S1 Table: Results of univariate regression between epidemic phase lag of London boroughs and census features, 1982-1990. Pearson's correlation coefficient and P-values are presented in the table. Regression coefficients with a significant association ($P < 0.05$) are shown in bold font
