# Supplementary material for: Dissecting recurrent waves of pertussis across the boroughs of London
Source: PLoS Comput Biol. 2022 Apr 14;18(4):e1009898. doi: 10.1371/journal.pcbi.1009898 (PMC9041754; doi:10.1371/journal.pcbi.1009898)
Supplement: S2 Table — (PDF) [file pcbi.1009898.s019.pdf]

| | *Regression coefficient* (*P*-value) | | | |
|---|---|---|---|---|
| Features | 1982 | 1986 | 1990 | Across years |
| No. households with No children under 16 | -3.3(0.164) | -2.0(0.453) | -5.3(0.094) | 0.08(0.863) |
| No. households with children aged 0 to 4 | 2.08(0.317) | 2.07(0.576) | 5.85(0.050) | 0.88(0.112) |
| No. households with children aged 5 to 16 | -6.2(0.054) | -3.4(0.427) | **-4.9(0.044)** | **-1.1(0.007)** |
| Born in Africa | -0.2(0.712) | -0.1(0.857) | 0.03(0.949) | -0.0(0.959) |
| Born in Caribbean | 0.83(0.316) | 0.62(0.598) | 1.17(0.285) | -0.2(0.460) |
| Born in India | -0.1(0.790) | 0.05(0.926) | -0.0(0.971) | 0.22(0.313) |
| Born in Pakistan | 0.22(0.534) | 0.01(0.979) | -0.3(0.314) | 0.00(0.960) |
| Pres. & Res. comm. estbls. | 0.20(0.751) | 1.24(0.293) | 1.60(0.073) | 0.50(0.069) |
| Households with $>$ 1.5 PPR | -1.4(0.064) | -1.5(0.109) | -0.8(0.124) | **-0.8(0.000)** |
| Not self contained houses | -0.2(0.709) | -0.2(0.832) | 2.15(0.214) | -0.1(0.605) |
| SEG 1-4 | 0.09(0.972) | 0.69(0.824) | -0.6(0.763) | -1.0(0.146) |
| SEG 8,9,12 | 0.33(0.884) | 0.62(0.821) | -2.9(0.285) | 0.24(0.75) |
| SEG 7-10 | 0.07(0.922) | 0.53(0.703) | -0.7(0.595) | 0.14(0.681) |
| SEG 11 | 0.04(0.955) | 0.10(0.919) | 2.09(0.257) | -0.3(0.295) |
| SEG 13-15 | 0.67(0.226) | 0.66(0.425) | 0.44(0.452) | **0.75(0.000)** |
| SEG 16-17 | 0.08(0.938) | -0.2(0.875) | -2.3(0.145) | -0.2(0.525) |
| Travel public | 3.29(0.146) | 1.58(0.262) | **3.05(0.048)** | **1.19(0.008)** |
| Travel other modes | 4.27(0.273) | -0.0(0.982) | 3.58(0.313) | -0.4(0.633) |
| Inland Area (Hectares) | 0.27(0.71) | 0.37(0.765) | 1.58(0.178) | -0.0(0.995) |
| Longitude | 0.50(0.435) | -0.0(0.935) | 0.40(0.630) | -0.3(0.102) |
| Latitude | 0.22(0.495) | 0.21(0.569) | 0.78(0.058) | 0.16(0.198) |

S2 Table: Results of multivariate regression between epidemic phase lag of London boroughs and census features, 1982-1990. Regression coefficients and $P$-values are presented in the table. Regression coefficients with a significant association ($P < 0.05$) are shown in bold font
