# Supplementary material for: Dissecting recurrent waves of pertussis across the boroughs of London
Source: PLoS Comput Biol. 2022 Apr 14;18(4):e1009898. doi: 10.1371/journal.pcbi.1009898 (PMC9041754; doi:10.1371/journal.pcbi.1009898)
Supplement: S3 Table — (PDF) [file pcbi.1009898.s020.pdf]

Feature	*Pearson's r* (*P*-value)
0-12mo	-0.0(0.917)
1-5yrs	0.01(0.915)
6-10yrs	0.04(0.805)
11-15yrs	0.08(0.644)
16-19yrs	0.10(0.552)
20+yrs	0.02(0.873)
Born in Africa	0.07(0.663)
Born in Caribbean	-0.0(0.975)
Born in India	-0.1(0.354)
Born in Pakistan	**-0.4(0.007)**
Pres. & Res. comm. estbls.	0.11(0.506)
Households with 1-1.5 PPR	0.00(0.986)
Households with ¿1.5 PPR	0.01(0.929)
Not self contained houses	0.08(0.643)
Travel public	-0.0(0.979)
Travel other modes	0.00(0.997)
Inland Area (Hectares)	0.08(0.619)
Longitude	0.13(0.459)
Latitude	0.03(0.868)
Employers, managers and professional workers	-0.0(0.957)
Intermediate occupations	0.01(0.951)
Lower Supervisory, and technical occupations	0.05(0.749)
Semi-routine	0.08(0.639)
Routine	0.09(0.609)
Never worked	0.08(0.628)
Full time student	0.08(0.628)
Ratio of cumulative unvaccinated children to total population	**0.40(0.019)**

S3 Table: Results of univariate regression between epidemic phase lag of London boroughs and census features, 2012. Pearson's correlation coefficient and P-values are presented in the table. Regression coefficients with a significant association ($P < 0.05$) are shown in bold font
