# Supplementary material for: Dissecting recurrent waves of pertussis across the boroughs of London
Source: PLoS Comput Biol. 2022 Apr 14;18(4):e1009898. doi: 10.1371/journal.pcbi.1009898 (PMC9041754; doi:10.1371/journal.pcbi.1009898)
Supplement: S1 Text — (PDF) [file pcbi.1009898.s021.pdf]

S1 Text. SHAP values

We use SHAP values to estimate the marginal contribution of each feature in the predicted value by the model for a given data instance, deemed as feature importance herein [1]. SHAP is rooted in "Shapley values", a concept from cooperative game theory first introduced in 1953 [2], which assigns a portion of the total payout to each player based on their marginal contribution to the coalition in a cooperative game setting. In the context of this paper, each feature is deemed as a player and the difference in predicted value by the model with and without a particular feature is considered as its importance.

Assume that effect (importance) of a given feature, $f_j \in F^p$, on the model prediction $J(x)$ for an individual input instance, $x^i = \{x_1^i, ..., x_p^i\}$, is desired, where F is set of all the features with p members. Given a set of features excluding f_j, $S \subseteq F \setminus \{f_j\}$, effect of f_j is the difference between the predicted value by the model with and without f_j:

$$J(x_{S \cup \{f_j\}}) - J(x_S) \tag{1}$$

Because effect of f_j also depends on the other features in S, eq. 1 must be calculated for all possible subsets $S \subseteq F \setminus \{f_j\}$. The shapely value, $\phi\{f_j, x_i\}$, is weighted average of all possible differences:

$$\phi\{f_j, x_i\} = \sum_{S \subseteq F \setminus \{f_j\}} \frac{|S|!\,(p - |S| - 1)!}{p!} \left(J(x_{S \cup \{f_j\}}) - J(x_S)\right) \tag{2}$$

The weighting term, $\frac{|S|!(p-|S|-1)!}{p!}$ accounts for all possible permutations of features added before and after f_j to the model. Number of terms in eq. 2 grows exponentially with number of features, and each term requires re-training the model with a different subset of features. Thus, in a high-dimensional feature space finding the exact solution of eq. 2 becomes computationally intensive. A sampling scheme can be used to find an approximation of eq. 2 [3]. We use the Kernel SHAP method [1] to find shapely values, which averts the need for repeated training of the model with different subset of features. Kernel SHAP approximates the contribution of each feature at an input instance, x^i, as follows:

- Generate sample vectors $z \in 0, 1^p$, where p is number of features. Each member in z represent a feature;
- Map z to the original feature space to obtain $x' = h(z)$. function $h_x : \{0, 1\}^p \rightarrow \mathbb{R}^p$, maps 1's in z to the value of the corresponding feature from the input instance, x^i, and maps 0's to value of the corresponding feature from from a random data instance, x^k;
- Apply the model on each x' to obtain $J(x')$. Since most models can not handle missing values, effect of missing features (0's in z) is approximated by integrating over samples from the input data, i.e. $J(x')$ is approximated with $E[J(x')|z_1]$, where z_1 is set of 1's in z;

- Assign a weight to each z using weighting kernel $\pi_{x'}(z)$:

$$\pi_x(z) = \frac{(p-1)}{\binom{p}{|z|}|z|(p-|z|)} \tag{3}$$

where $|z|$ is the number of 1's in z;

- Assuming $g(z)$, the local explanation model for $J(x')$ at a input instance $x' = h(z)$, to be a linear function of z parameterized by shapley values ϕ_j:

$$g(z) = \phi_0 + \sum_{j=1}^{p} \phi_j z_j \tag{4}$$

shapely values, ϕ_j, are obtained by fitting the weighted linear model given in eq. 4 by minimizing the following loss function:

$$\underset{g}{\operatorname{argmin}}\, L(J, g, \pi_x) \tag{5}$$

$$L(J, g, \pi_x) = \sum_{z \in Z} [J(h_x(z)) - g(z)]^2 \pi_x(z) \tag{6}$$

The loss function $L(J, g, \pi_x)$, and weighting kernel $\pi_x(z)$ are chosen such that solution to 5 recovers the shapely values given by 2 (see supplementary information of [1] for proof).

References

1. Lundberg SM, Allen PG, and Lee SI. A Unified Approach to Interpreting Model Predictions. 31st Conference on Neural Information Processing Systems (NIPS 2017). 2017
2. Shapley LS. A value for n-person games. The Shapley Value: Essays in Honor of Lloyd S. Shapley. Ed. by Roth AE. Cambridge University Press, 1988 :31–40. DOI: 10.1017/CBO9780511528446.003
3. Štrumbelj E and Kononenko I. Explaining prediction models and individual predictions with feature contributions. Knowledge and Information Systems 2014 Nov; 41:647–65. DOI: 10.1007/s10115-013-0679-x
